# Effects of thiamethoxam insecticide on sugarcane plant growth under chemical ripening at early and late harvest

**DOI:** 10.3389/fpls.2025.1558071

**Published:** 2025-06-17

**Authors:** Deise de Paula Silva, Josiane Viveiros, Lucas Moraes Jacomassi, Marcela Pacola, Letusa Momesso, Gabriela Ferraz de Siqueira, Jorge Martinelli Martello, Rodrigo Foltran, Rogério Peres Soratto, Leila Luci Dinardo-Miranda, Carlos Alexandre Costa Crusciol

**Affiliations:** ^1^ Sugarcane Research Unit, United States Department of Agriculture–Agricultural Research Service (USDA-ARS), Houma, LA, United States; ^2^ São Paulo State University (UNESP), College of Agricultural Sciences, Department of Crop Science, Botucatu, São Paulo, Brazil; ^3^ Federal University of Goiás (UFG), School of Agriculture, Department of Agriculture, Goiânia, Goiás, Brazil; ^4^ Agronomic Institute of Campinas (IAC), Sugarcane Center, Ribeirão Preto, Brazil

**Keywords:** biostimulant, phytotonic effect, plant regulators, *Saccharum* spp., sugarcane yield

## Abstract

Chemical ripeners are applied to ensure the quality of the final product in sugarcane production, especially under unfavorable conditions for sucrose accumulation. In addition, bioactivators such as the insecticide thiamethoxam can stimulate plant development. Thus, the application of thiamethoxam to sugarcane regrowth associated with ripener may have phytotonic effects and improve sugarcane quality and yield. The aim of this study was to understand the effects of thiamethoxam foliar application to sugarcane ratoon treated with trinexapac-ethyl as a ripener. Four management strategies (treatments) were introduced and tested in six field experiments conducted across the early and late harvest seasons: no chemical application (control), application of 250 g a.i ha^-1^ trinexapac-ethyl (0.4 L ha^-1^ of commercial product) as a ripener, application of thiamethoxam 100 g a.i ha^-1^ (0.4 kg ha^-1^ of commercial product) as a bioactivator, and application of ripener and bioactivator. Thiamethoxam application increased stalk yield by 14 Mg ha^-1^ compared with the control, and joint application with ripener increased sugar yield by up to 3 Mg ha^-1^ due to an increase in total recoverable sugar (TRS) of up to 11% compared with the control (139.9 kg Mg^-1^). The increases in biomass in response to thiamethoxam application increased potential energy production (MWh) by 16.8% compared with the treatments without insecticide. The integration of thiamethoxam into sugarcane management enhanced yield, biomass, and energy-related traits without compromising technological quality. When combined with trinexapac-ethyl, it increased sugar yield per hectare. These benefits point to improved land-use efficiency. However, given its classification as a neonicotinoid, further studies are needed to assess long-term safety. Such research is key to aligning productivity with sustainability in sugarcane systems.

## 1 Introduction

Sugarcane harvested in the early season (March-April) or late season (October-December) often fails to meet industrial quality standards, as sucrose content peaks in the middle of the harvest season (June–August) (Caputo et al., 2008). Sucrose accumulation is influenced by environmental conditions, particularly temperature and water availability, and when these are not favorable, chemical ripeners like trinexapac-ethyl are used to optimize ripening (Dinardo-miranda, 2005; Cardozo et al., 2014, 2020). Chemical ripeners are plant growth regulators that alter plant morphology and physiology to produce quantitative and qualitative changes in crop yield (Leite et al., 2015a). Trinexapac-ethyl enhances sucrose accumulation by inhibiting gibberellic acid systhesis, improving sugar yield without negatively impacting juice quality or crop weight (Resende et al., 2000; Zhao et al., 2023).

Beyond ripeners, certain insecticides and fungicides, such as thiamethoxam, have been recognized for their biostimulant effects (Martins et al., 2012). When applied at low doses, these compounds can stimulate vegetative growth even under adverse environmental conditions (Casillas V. et al., 1986). In addition to pest control, thiamethoxam has been shown to enhance plant metabolism, including amino acid synthesis, precursors for hormone production, as well as root development and nutrient uptake, contributing to improved plant establishment and increased biomass accumulation (Castro et al., 2008; Ford et al., 2010; Martins et al., 2012). These physiological benefits are also associated with increased tillering, stalk elongation, and ultimately, higher sugarcane yields (Silva et al., 2022; de Paula Silva et al., 2023). Moreover, thiamethoxam promotes photoassimilate accumulation in storage organs and stimulates the expression of stress-responsive genes, further enhancing raw material quality (Silva et al., 2009).

Although the positive impact of chemical ripeners on sugarcane production is well known (Legendre, 1975; van Heerden, 2014a; Leite et al., 2015b, 2015a; van Heerden et al., 2015), some studies evaluated the bioregulatory action of thiamethoxam on sugarcane (Martins et al., 2012), especially in the presence of potential deleterious effects of ripener use. Thiamethoxam application improves plant vigor in the early development stages as well as the response to stresses (Macedo and Castro, 2011; Martins et al., 2012), suggesting that it indirectly increases endogenous hormone synthesis.

Given its phytotonic effects, thiamethoxam may counteract some of the physiological constraints imposed by ripeners such as trinexapac-ethyl, offering a complementary strategy for improved sugarcane crop management [(Silva et al., 2022; de Paula Silva et al., 2023)]. Considering the economic importance of sugarcane and the widespread use of both pesticides and ripeners, it is essential to evaluate whether the sequential application of thiamethoxam following trinexapac-ethyl treatment can optimize agronomic practices and improve land use efficiency agroindustrial performance, economic return, and production efficiency.

In this context, the study tested the hypothesis that bioactivator, thiametoxam, can mitigate the adverse effects of using trinexapac-ethyl as a ripener in sugarcane management. To investigate this, we evaluated a management strategy based on the foliar application of thiamethoxam to ratoon sugarcane previously treated with trinexapac-ethyl. The objective was to assess physiological and agronomic responses through sucrose accumulation, stalk yield, and biomass production, under both early and late harvest conditions.

## 2 Materials and methods

### 2.1 Site description

**Figure 1:**
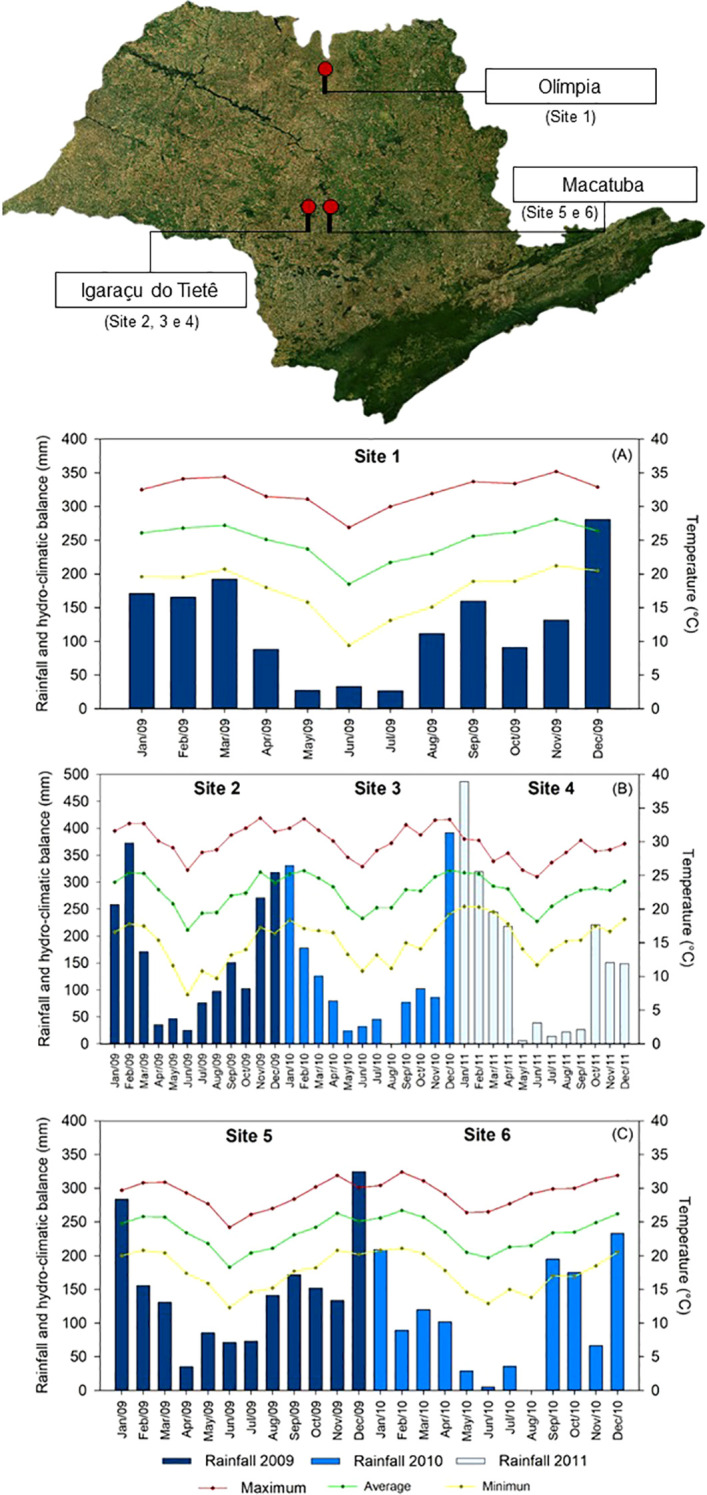
Rainfall (mm) and average monthly temperature (°C) during the experiment conduction in the Site 1 **(A)**, Sites 2, 3 and 4 **(B)** and Sites 5 and 6 **(C)**.

Six experimental trials were conducted during the early and late harvest seasons of sugarcane (*Saccharum* spp. hybrids) across three locations in São Paulo State, Brazil. The early season trials were performed in Olimpia (20°46’96 “S and 49°49’15” W) (site 1) and in Igaraçu do Tietê (22°33’18 “S and 48°3’51’’W) (site 2), (site 3) and (site 4). Late season trials were conducted in Macatuba (22°30’08’S and 48°42’41’’W) (sites 5 and 6) (**Figure 1**). Site 1 has a Cwa (Köppen) climate with an average annual temperature of 23.4°C and average rainfall of 1,285 mm, and the soil at this site is classified as a eutrophic red, yellow Argisoil with medium/clay texture (USDA - Soil Survey Staff, and STAFF, S. S, 2014). Sites 2, 3 and 4 have an Aw (Köppen) climate with an average annual temperature of 21.6°C and average rainfall of 1,344 mm, and the soil at these sites is classified as a Eutrophic Purple Latosol with clayey texture (USDA - Soil Survey Staff, and STAFF, S. S, 2014). Sites 5 and 6 have an Aw (Köppen) climate with an average annual temperature of 25°C and average rainfall of 1,244 mm; the soil is classified as a Eutrophic Purple Latosol with clayey texture (USDA - Soil Survey Staff, and STAFF, S. S, 2014). Rainfall and air temperature data are shown in **Figure 1**.

### 2.2 Experimental design and treatment application

**Figure 2:**
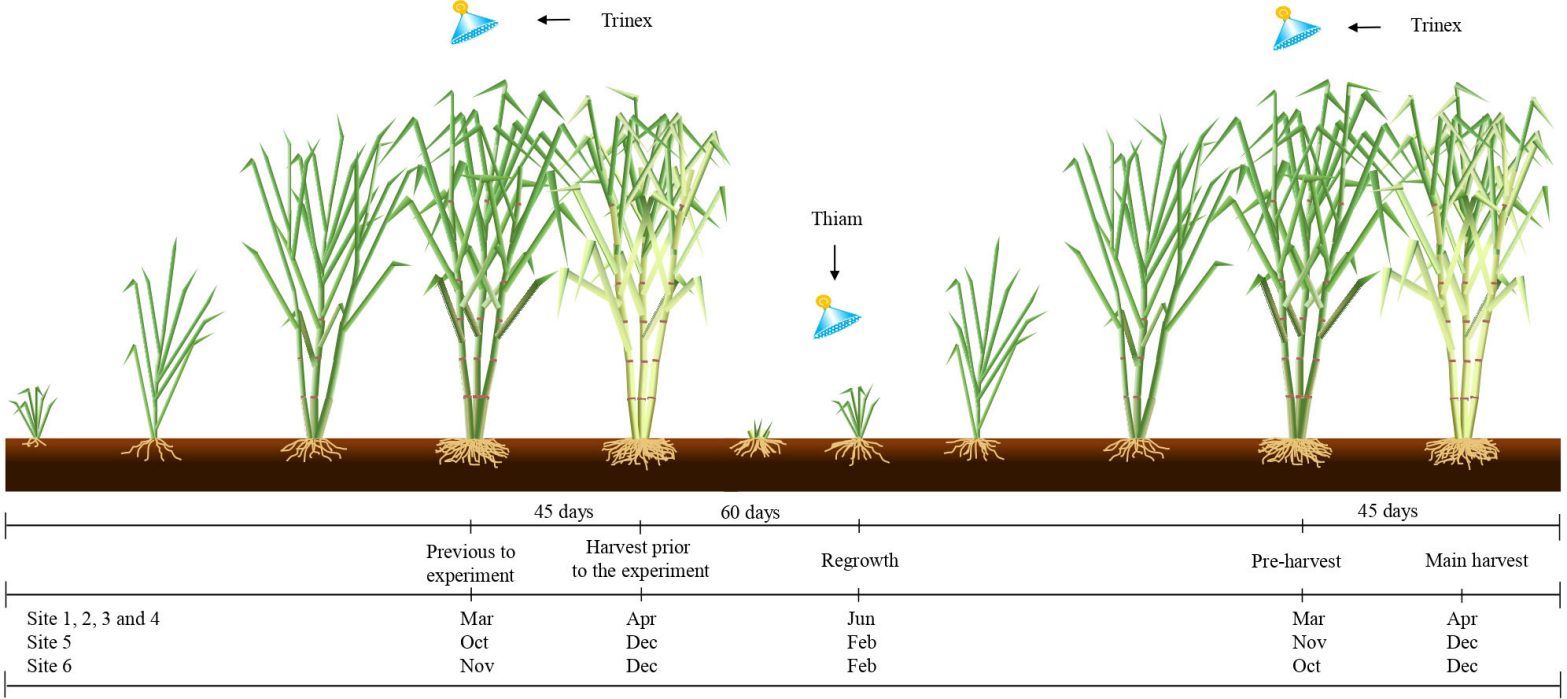
Schematic diagram of chemical applications when applying ripener (Trinex: trinexapac-ethyl application) at 45 days before sugarcane harvest, and bioactivator (Thiam: thiamethoxam application) at 60 days after sugarcane regrowth stage.

The early-ripening variety RB855453 were used for early harvest season and the late-ripening variety SP803280 were used for the late harvest season. The experimental design was randomized, within each site, and comprised four treatments with five repetitions. The treatments were as follows: (i) control (C), no ripener or bioactivator was applied to sugarcane; (ii) thiamethoxam (Thiam), bioactivator was applied at 60 days after the beginning of sugarcane regrowth; (iii) trinexapac-ethyl (Trinex), ripener was applied at 45 days previously the harvest before the experiment and 45 days prior the main harvest; and (iv) thiamethoxam + trinexapac-ethyl (Thiam+Trinex), thiamethoxam and trinexapac-ethyl were applied as described in treatments ii and iii (**Figure 2**). The applied doses of thiamethoxam and trinexapac-ethyl were 100 and 250 g i.a. ha^-1^, respectively. Thiamethoxam is traded as Actara^®^ (Syngenta Proteção de Cultivos Ltda., São Paulo, SP). On the other hand, trinexapac-ethyl is traded as Moddus^®^ (Syngenta Crop Protection Ltd., São Paulo, SP) for foliar application. Treatment application dates are specified in **Figure 2**. The plots consisted of 8 sugarcane rows 10 m long and inter-row spacing of 1.4 m.

The ripener was applied in March and in October/December in the early and late season respectively as recommended by the manufacturer, i.e., approximately 45 days prior the sugarcane harvest before the experiment setup in all treatments with trinexapac-ethyl (**Figure 2**). As only ripener was applied at this time, no evaluations of the plots were performed. After subsequent regrowth, the experiment was carried out by applying thiamethoxam 60 days after the regrowth start and trinexapac-ethyl 45 days before the main harvest (**Figure 2**). All applications were carried out under adequate weather conditions at the beginning of the day. Spraying was performed using equipment equipped with a CO_2_ cylinder. Thiamethoxam application was in a directed jet in the sugarcane rows; for ripener, a T-shaped manual bar 3 m length was used reaching two rows simultaneously. The bar had 6 nozzles of type AXI 11002 spaced 0.5 m apart. Spraying was performed at a pressure of 344 kPa in a 100 L ha-1 water volume.

Applications were carried out at the specified times based on the phenological stages of the sugarcane crop and in accordance with technical recommendations from the manufacturers. Thiamethoxam was applied during the active tillering phase, a critical stage for crop establishment, as it determines stalk density and contributes to initial plant vigor. The application of systemic insecticides at this stage supports plant health by controlling early-season pests and, under certain conditions, can trigger beneficial physiological responses, such as enhanced root development and improved tolerance to abiotic stress (Pereira et al., 2010; Silva et al., 2022; Syngenta Proteção de Cultivos Ltda, 2023). In turn, trinexapac-ethyl was applied at the onset of the maturation phase, a period marked by reduced vegetative growth and increased sucrose accumulation. Its use is intended to limit excessive shoot growth and promote the remobilization of photoassimilates to the stalks, thereby enhancing sugar concentration and improving the technological quality of the raw material (Leite et al., 2009a; De Lima et al., 2019; Syngenta Proteção de Cultivos Ltda, 2024).

Pest assessments were carried out before the experiments, and no pest problems were revealed in the chosen fields at the experimental sites. All the experimental field management were made according to each site recommendations, and no other products with bioactivator characteristics were used.

### 2.3 Sugarcane measurements

#### 2.3.1 Biometric and quality parameters

Evaluations were carried out 40 days after ripener application, i.e., at the ripening stage of sugarcane. A sample of 20 stalks randomly collected from each plot was used to assess sugarcane biometric parameters and biomass production. Plant height (m) was measured with graduated ruler and the stalks present in 6 m of the two central rows of plants were counted to determine the number of stalks per meter.

To verify the raw material technological quality, the same sample of 20 stalks used to measure biometric parameters was cut at the apical bud height, defoliated and sent to the PCTS laboratory of the mill for determination of fiber % (dry water-insoluble matter); sucrose (%) (sucrose concentration in the fresh weight); reducing sugars (RS %) (reducing substances in cane and sugar products calculated as invert sugar, predominantly hexoses), purity % (sucrose content in the total solids content); total reducing sugar (TRS) (all forms of sugars in form of reducing or inverted sugars) according to the Sucrose Content-Based Sugarcane Payment System methodology defined in accordance with semiannual updates for the technological evaluations of Consecana described by (Fernandes, 2011).


TRS=(Sucrose concentration×9.5263)+(RS×9.05)

#### 2.3.2 Stalk and sugar yield

Stalk yield was determined by harvesting each plot mechanically, weighing the stalks with an electronic load cell, and extrapolating to Mg ha ^-1^. Then, sugar yield (Mg ha^-1^) calculation was carried out with the stalk yield (Mg ha^-1^) value multiplied by TRS and dividing by 100.


$$\text{Sugar yield }({\text{Mg ha}}^{-1})=({\text{TRS (kg Mg}}^{-1})\times \text{Stalk yield }({\text{Mg ha}}^{-1})/1000$$

#### 2.3.3 Biomass yield and energy production

To calculate bagasse at 50% moisture the results for fiber and stalk yield were used.


$$\text{Bagasse}=\frac{fiber\;\times \;stalk\;yield}{100}$$

Trash yield was determined considering 140 kg of trash per Mg of stalk and 60% collection from the soil surface (Hassuani, 2005).


Trash=Stalk yield×0.14×0.6

The lack of moisture accumulation at the base of the plants by the absence of soil cover due to the trash residue collection from the plots minimized pest problems, especially leafhoppers (*Mahanarva fimbriolata*). Energy production was determined considering that 1 Mg of trash has 4.96 MWh of primary energy and 1 Mg of bagasse has 4.94 MWh of primary energy (1 MWh = 3,600.00 MJ) (Hassuani, 2005).


Energy=bagasse×4.94+trash×4.96

## 3 Data analysis

All data were first tested for normality using the Shapiro–Wilk test and for homogeneity of variances using Levene’s test, both performed in Minitab 19. Subsequently, analysis of variance (ANOVA) was conducted using the F-test at a significance level of *p* ≤ 0.05. The degrees of freedom were 4 for blocks, 3 for treatments, and 12 for the residual, totaling 19 degrees of freedom. Means were compared using Fisher’s protected least significant difference (LSD) test at the same significance level. All statistical analyses were performed using the SISVAR software. We built a heatmap of the Pearson correlation coefficients (p ≤ 0.05) among the measured variables.

## 4 Results

### 4.1 Biometric and quality parameters

Thiamethoxam applied alone increased plant height at sites 1, 2, and 6 (**Table 1**), although it did not differ significantly from the control. With the exception of fiber content, all qualitative parameters showed significant effects (p < 0.05) (**Table 1**). The application of trinexapac-ethyl, either alone or in combination with thiamethoxam, improved the quality of raw material for industrial use, with average juice purity exceeding 80% across all sites. On the other hand, the application of trinexapac-ethyl and thiamethoxam + trinexapac-ethyl reduced RS compared to the control and thiamethoxam treatments at all locations.

**Table 1:** Fiber, purity, reducing sugars (RS) and stalk height in sugarcane receiving the application of thiamethoxam as bioactivator and trinexapac-ethyl as ripener at ratoon regrowth.

Treatments†	Early harvest season				Late harvest season	
	Site 1	Site 2	Site 3	Site 4	Site 5	Site 6
	Stalk height (m)					
Control	2.39 ab	2.43 ab	2.45 a	2.33 a	2.31 a	2.32 ab
Thiamethoxam (Thiam)	2.45 a	2.50 a	2.56 a	2.37 a	2.36 a	2.45 a
Trinexapac-ethyl (Trinex)	2.04 c	2.24 b	2.38 a	2.26 a	2.22 a	2.20 b
Thiam+Trinex	2.25 b	2.28 b	2.44 a	2.27 a	2.30 a	2.28 b
*F probability*	*0.049*	*0.042*	*0.188*	*0.202*	*0.156*	*0.033*
	Fiber (%)					
Control	12.3 a	12.0 a	12.3 a	11.0 a	13.2 a	14.5 a
Thiamethoxam (Thiam)	12.0 a	12.2 a	12.5 a	10.8 a	13.1 a	14.4 a
Trinexapac-ethyl (Trinex)	12.5 a	12.0 a	12.4 a	11.4 a	13.6 a	14.8 a
Thiam+Trinex	11.9 a	12.5 a	12.7 a	11.6 a	13.9 a	14.9 a
*F probability*	*0.158*	*0.203*	*0.338*	*0.174*	*0.15*	*0.189*
	Purity (%)					
Control	87.2 b	86.8 b	86.8 b	80.8 b	82.2 b	85.6 b
Thiamethoxam (Thiam)	87.3 b	87.1 b	87.0 b	80.9 b	82.6 b	85.4 b
Trinexapac-ethyl (Trinex)	89.5 a	90.1 a	89.2 a	85.1 a	85.7 a	87.9 a
Thiam+Trinex	89.2 a	89.5 a	89.6 a	85.4 a	85.9 a	88.8 a
*F probability*	*0.048*	*0.036*	*0.044*	*0.025*	*0.021*	*0.039*
	Reducing sugars (%)					
Control	0.51 a	0.51 a	0.52 a	0.70 a	0.61 a	0.58 a
Thiamethoxam (Thiam)	0.50 a	0.53 a	0.53 a	0.74 a	0.63 a	0.58 a
Trinexapac-ethyl (Trinex)	0.45 b	0.45 b	0.46 b	0.58 b	0.54 b	0.50 b
Thiam+Trinex	0.45 b	0.47 b	0.45 b	0.56 b	0.55 b	0.51 b
*F probability*	*0.012*	*0.023*	*0.014*	*0.037*	*0.049*	*0.042*

†Stalk height, fiber, purity and reducing sugar of sugarcane receiving the application of thiamethoxam as bioactivator and trinexapac-ethyl as ripener at ratoon regrowth. Treatments of chemical application strategy mean control, no-chemical applications; Thiam, thiamethoxam applied at 60 after sugarcane regrowth stage; Trinex, trinexapac-ethyl applied at 45 days before sugarcane harvest; and Thiam+Trinex, thiamethoxam and trinexapac-ethyl applied as treatments Thiam (60 after sugarcane regrowth stage) and Trinex (45 days before sugarcane harvest). All data show the means of four replicates. Different letters indicate significant (p < 0.05) differences between treatments by LSD test.

### 4.2 Stalk and sugar yield

**Figure 3:**
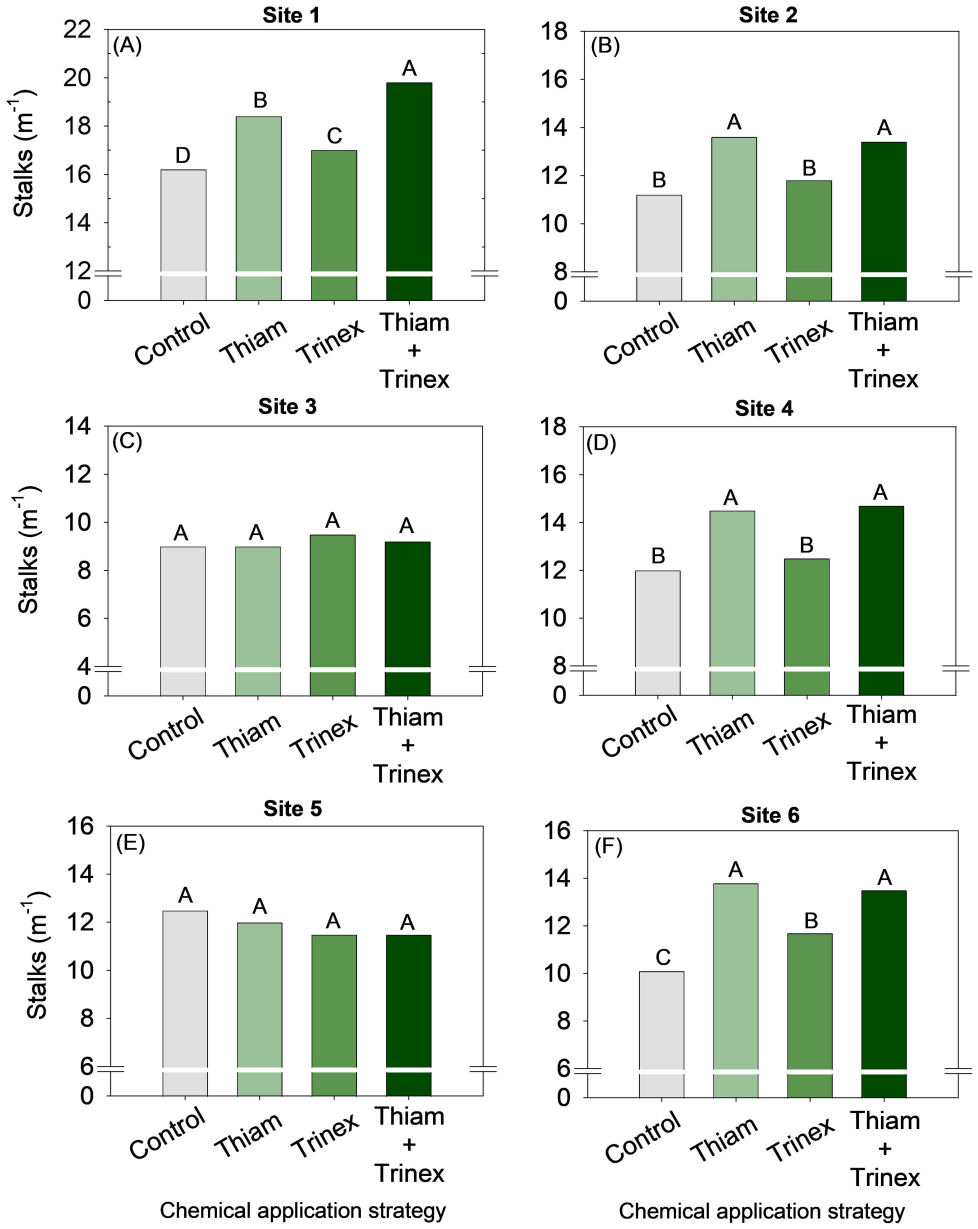
Stalk number of sugarcane receiving the application of thiamethoxam as bioactivator and trinexapac-ethyl as ripener at Site 1 **(A)**, Site 2 **(B)**, Site 3 **(C)**, Site 4 **(D)**, Site 5 **(E)** and Site 6 **(F)**. Treatments of chemical application strategy mean control, no-chemical applications; Thiam, thiamethoxam applied at 60 after sugarcane regrowth stage; Trinex, trinexapac-ethyl applied at 45 days before sugarcane harvest; and Thiam+Trinex, thiamethoxam and trinexapac-ethyl applied as treatments Thiam (60 after sugarcane regrowth stage) and Trinex (45 days before sugarcane harvest). All data show the means of four replicates. Different letters indicate significant (p < 0.05) differences between treatments by LSD test.

**Figure 4:**
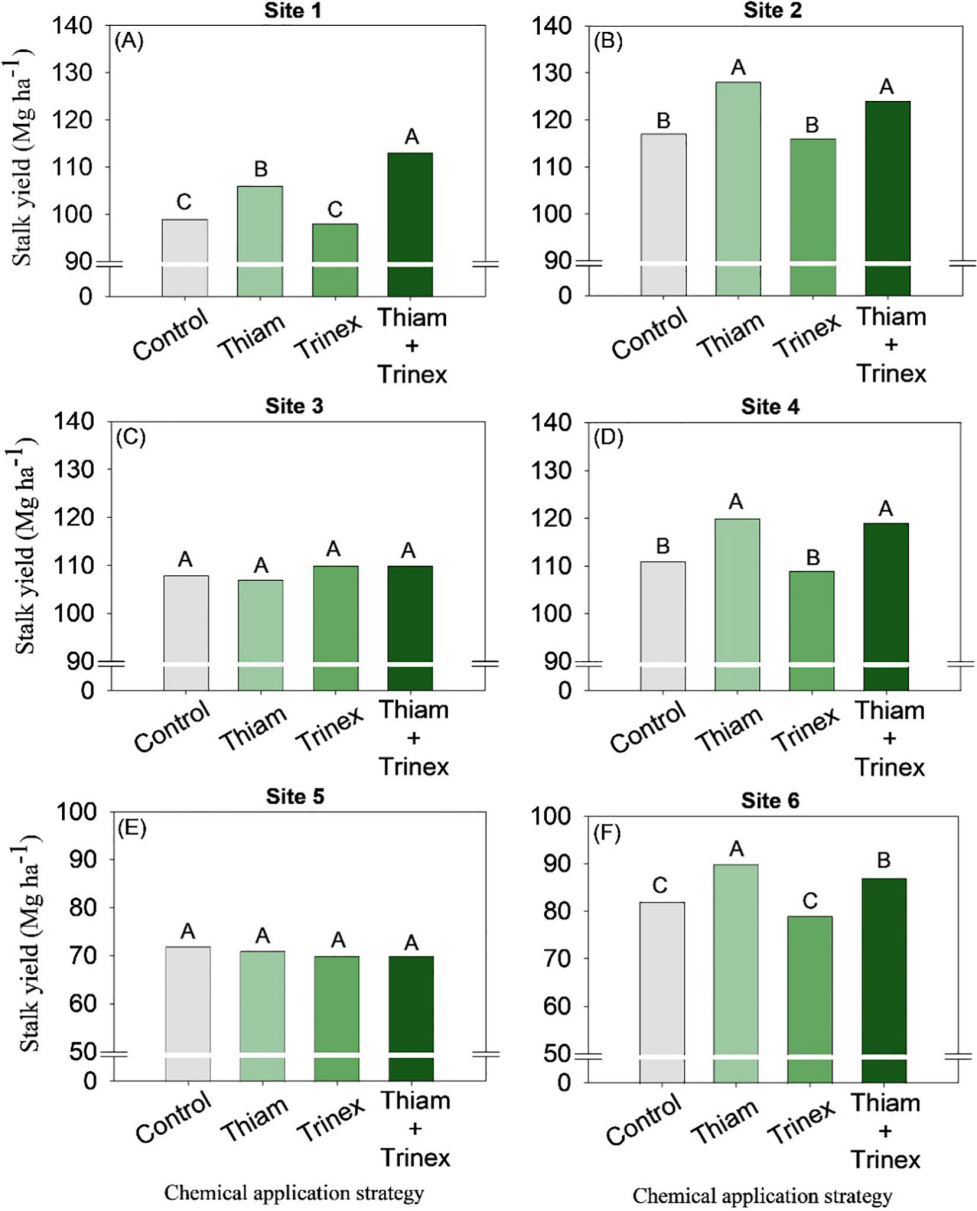
Stalk yield of sugarcane receiving the application of thiamethoxam as bioactivator and trinexapac-ethyl as ripener at Site 1 **(A)**, Site 2 **(B)**, Site 3 **(C)**, Site 4 **(D)**, Site 5 **(E)** and Site 6 **(F)**. Treatments of chemical application strategy mean control, no-chemical applications; Thiam, thiamethoxam applied at 60 after sugarcane regrowth stage; Trinex, trinexapac-ethyl applied at 45 days before sugarcane harvest; and Thiam+Trinex, thiamethoxam and trinexapac-ethyl applied as treatments Thiam (60 after sugarcane regrowth stage) and Trinex (45 days before sugarcane harvest). All data show the means of four replicates. Different letters indicate significant (p < 0.05) differences between treatments by LSD test.

Overall, the application of thiamethoxam and thinexapac-ethyl + thiamethoxam increased stalk yield by an average of 2 stalks m^-1^ compared to the control (**Figure 3**). At sites 3 and 5, no significant differences were observed among treatments (**Figures 3C, E**). In general, for early harvest sugarcane (sites 1, 2, and 4), the application of thiamethoxam + trinexapac-ethyl significantly increased stalk yield (p < 0.05) (**Figure 4**). The highest increase, approximately 14 Mg ha^-1^, was observed at site 1 compared to the control (99 Mg ha^-1^) (**Figure 4A**). On the other hand, for late-harvest sugarcane, only site 6 showed an increase with the application of Thiam alone, resulting in a gain of approximately 8 Mg ha^-1^ compared to the control (82 Mg ha^-1^) (**Figure 4F**).

**Figure 5:**
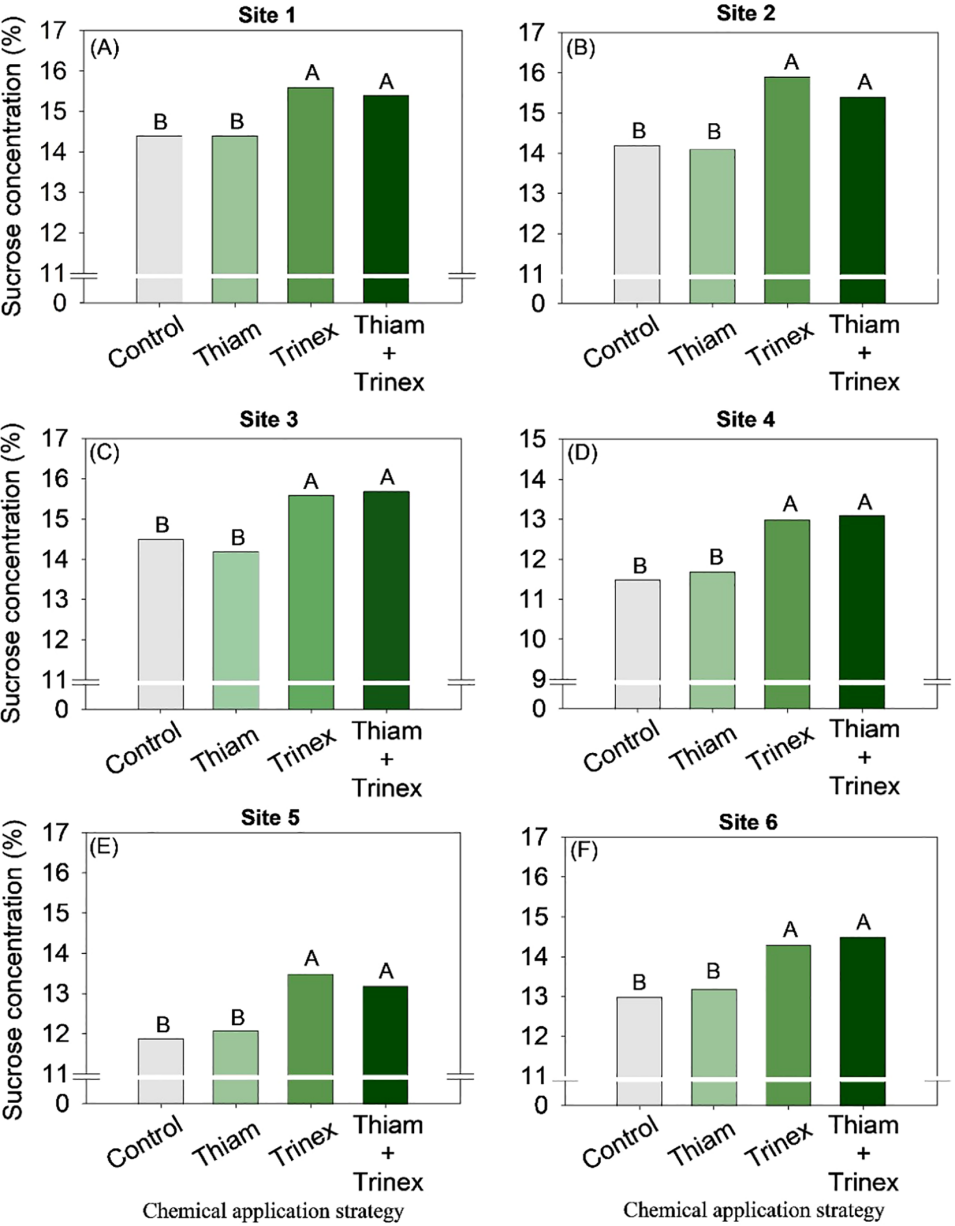
Sucrose concentration of sugarcane receiving the application of thiamethoxam as bioactivator and trinexapac-ethyl as ripener at Site 1 **(A)**, Site 2 **(B)**, Site 3 **(C)**, Site 4 **(D)**, Site 5 **(E)** and Site 6 **(F)**. Treatments of chemical application strategy mean control, no-chemical applications; Thiam, thiamethoxam applied at 60 after sugarcane regrowth stage; Trinex, trinexapac-ethyl applied at 45 days before sugarcane harvest; and Thiam+Trinex, thiamethoxam and trinexapac-ethyl applied as treatments Thiam (60 after sugarcane regrowth stage) and Trinex (45 days before sugarcane harvest). All data show the means of four replicates. Different letters indicate significant (p < 0.05) differences between treatments by LSD test.

**Figure 6:**
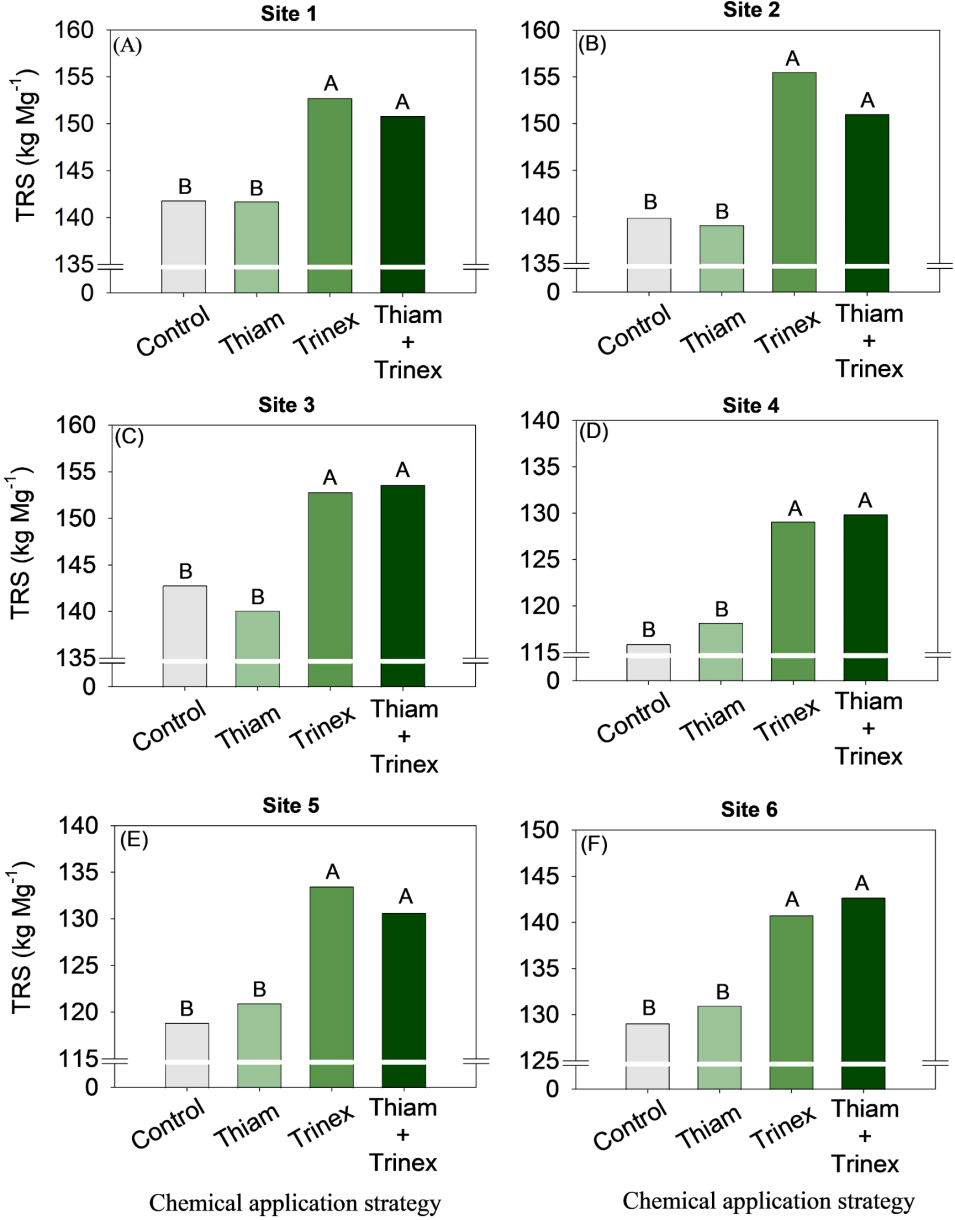
Total reducing sugars of sugarcane receiving the application of thiamethoxam as bioactivator and trinexapac-ethyl as ripener at Site 1 **(A)**, Site 2 **(B)**, Site 3 **(C)**, Site 4 **(D)**, Site 5 **(E)** and Site 6 **(F)**. Treatments of chemical application strategy mean control, no-chemical applications; Thiam, thiamethoxam applied at 60 after sugarcane regrowth stage; Trinex, trinexapac-ethyl applied at 45 days before sugarcane harvest; and Thiam+Trinex, thiamethoxam and trinexapac-ethyl applied as treatments Thiam (60 after sugarcane regrowth stage) and Trinex (45 days before sugarcane harvest). All data show the means of four replicates. Different letters indicate significant (p < 0.05) differences between treatments by LSD test.

**Figure 7:**
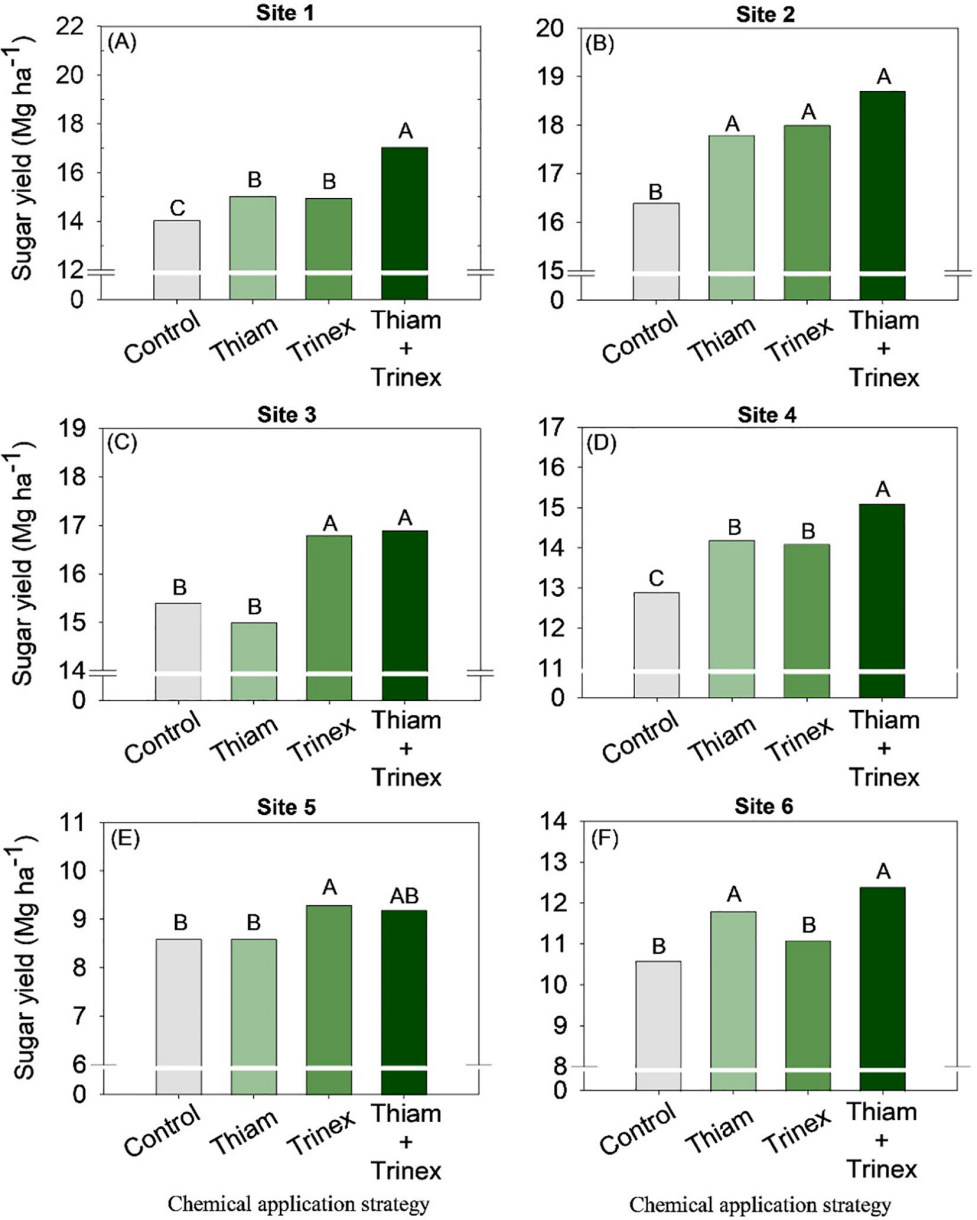
Sugar yield of sugarcane receiving the application of thiamethoxam as bioactivator and trinexapac-ethyl as ripener at Site 1 **(A)**, Site 2 **(B)**, Site 3 **(C)**, Site 4 **(D)**, Site 5 **(E)** and Site 6 **(F)**. Treatments of chemical application strategy mean control, no-chemical applications; Thiam, thiamethoxam applied at 60 after sugarcane regrowth stage; Trinex, trinexapac-ethyl applied at 45 days before sugarcane harvest; and Thiam+Trinex, thiamethoxam and trinexapac-ethyl applied as treatments Thiam (60 after sugarcane regrowth stage) and Trinex (45 days before sugarcane harvest). All data show the means of four replicates. Different letters indicate significant (p < 0.05) differences between treatments by LSD test.

The application of trinexapac-ethyl, either alone or in combination with Thiam, increased sucrose concentration at all sites (**Figure 5**). In early-harvest conditions (**Figures 5A–D**), both treatments resulted in an average increase of 10.3% compared to the control (13.6%). In late-harvest conditions, the increase was approximately 13% compared to the control (12.5%) (**Figures 5E, F**).Similarly, TRS increased at all sites when trinexapac-ethyl was used, whether alone or in association with thiamethoxam (Trinex or Thiam+Trinex) (**Figure 6**). This increase was most notable at site 2 (early season), with a gain of approximately 11% in trinexapac-ethyl compared with the control (139.9 kg Mg^-1^). As sugar yield is the product of TRS and stalk yield, this variable was also affected by the treatments at all sites (**Figure 7**). Overall, at the early season, the ripener applied in combination with thiamethoxam resulted in higher sugar yield across all sites, with the most notable increase observed at site 1, where the increase was 3 Mg ha^-1^ compared to the control (14 Mg ha^-1^). In the late harvest, results varied between the two sites. At site 5 (**Figure 7E**), the application of trinexapac-ethyl alone resulted in the highest sugar yield (9.3 Mg ha^-1^), although it did not differ from the combination with thiamethoxam (9.2 Mg ha^-1^). The average gain compared to the control (8.6 Mg ha^-1^) was approximately 7%. At site 6 (**Figure 7F**), both thiamethoxam applied alone (13.6 Mg ha^-1^) and in combination with trinexapac-ethyl (13.4 Mg ha^-1^) led to higher sugar yields, with no significant difference between them. The average increase over the control was approximately 8%.

### 4.3 Biomass yield and energy production

**Figure 8:**
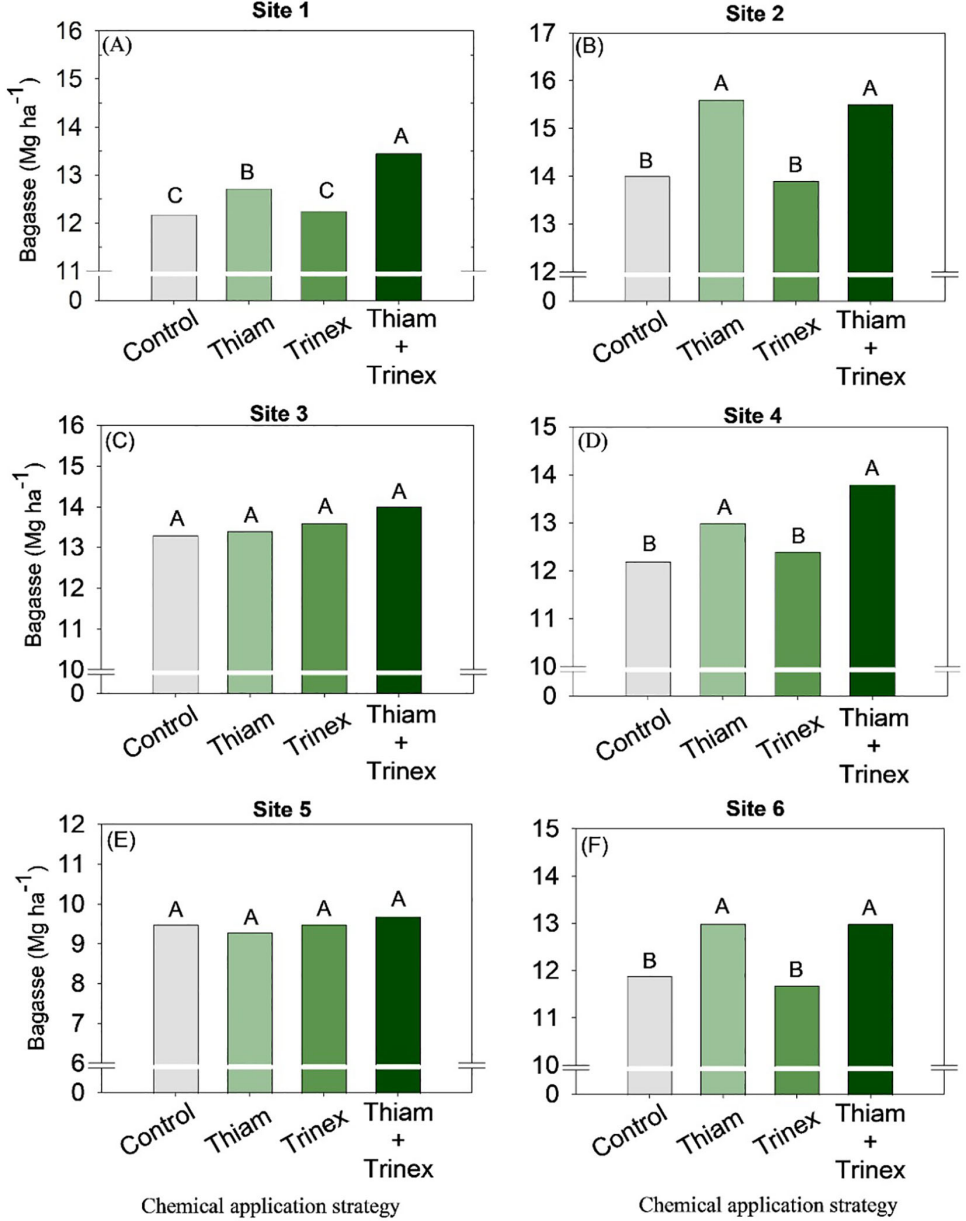
Bagasse of sugarcane receiving the application of thiamethoxam as bioactivator and trinexapac-ethyl as ripener at Site 1 **(A)**, Site 2 **(B)**, Site 3 **(C)**, Site 4 **(D)**, Site 5 **(E)** and Site 6 **(F)**. Treatments of chemical application strategy mean control, no-chemical applications; Thiam, thiamethoxam applied at 60 after sugarcane regrowth stage; Trinex, trinexapac-ethyl applied at 45 days before sugarcane harvest; and Thiam+Trinex, thiamethoxam and trinexapac-ethyl applied as treatments Thiam (60 after sugarcane regrowth stage) and Trinex (45 days before sugarcane harvest). All data show the means of four replicates. Different letters indicate significant (p < 0.05) differences between treatments by LSD test.

**Figure 9:**
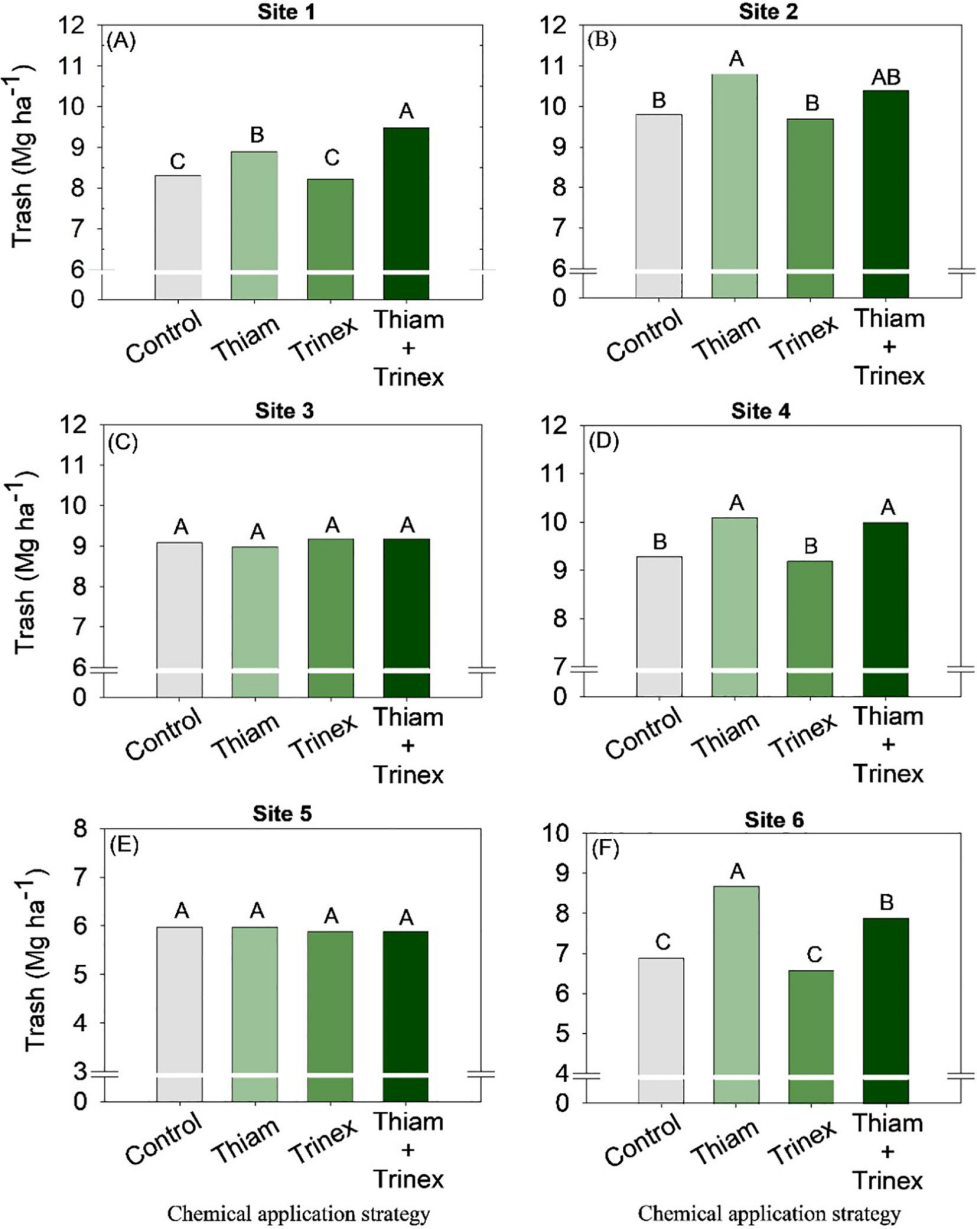
Trash of sugarcane receiving the application of thiamethoxam as bioactivator and trinexapac-ethyl as ripener at Site 1 **(A)**, Site 2 **(B)**, Site 3 **(C)**, Site 4 **(D)**, Site 5 **(E)** and Site 6 **(F)**. Treatments of chemical application strategy mean control, no-chemical applications; Thiam, thiamethoxam applied at 60 after sugarcane regrowth stage; Trinex, trinexapac-ethyl applied at 45 days before sugarcane harvest; and Thiam+Trinex, thiamethoxam and trinexapac-ethyl applied as treatments Thiam (60 after sugarcane regrowth stage) and Trinex (45 days before sugarcane harvest). All data show the means of four replicates. Different letters indicate significant (p < 0.05) differences between treatments by LSD test.

**Figure 10:**
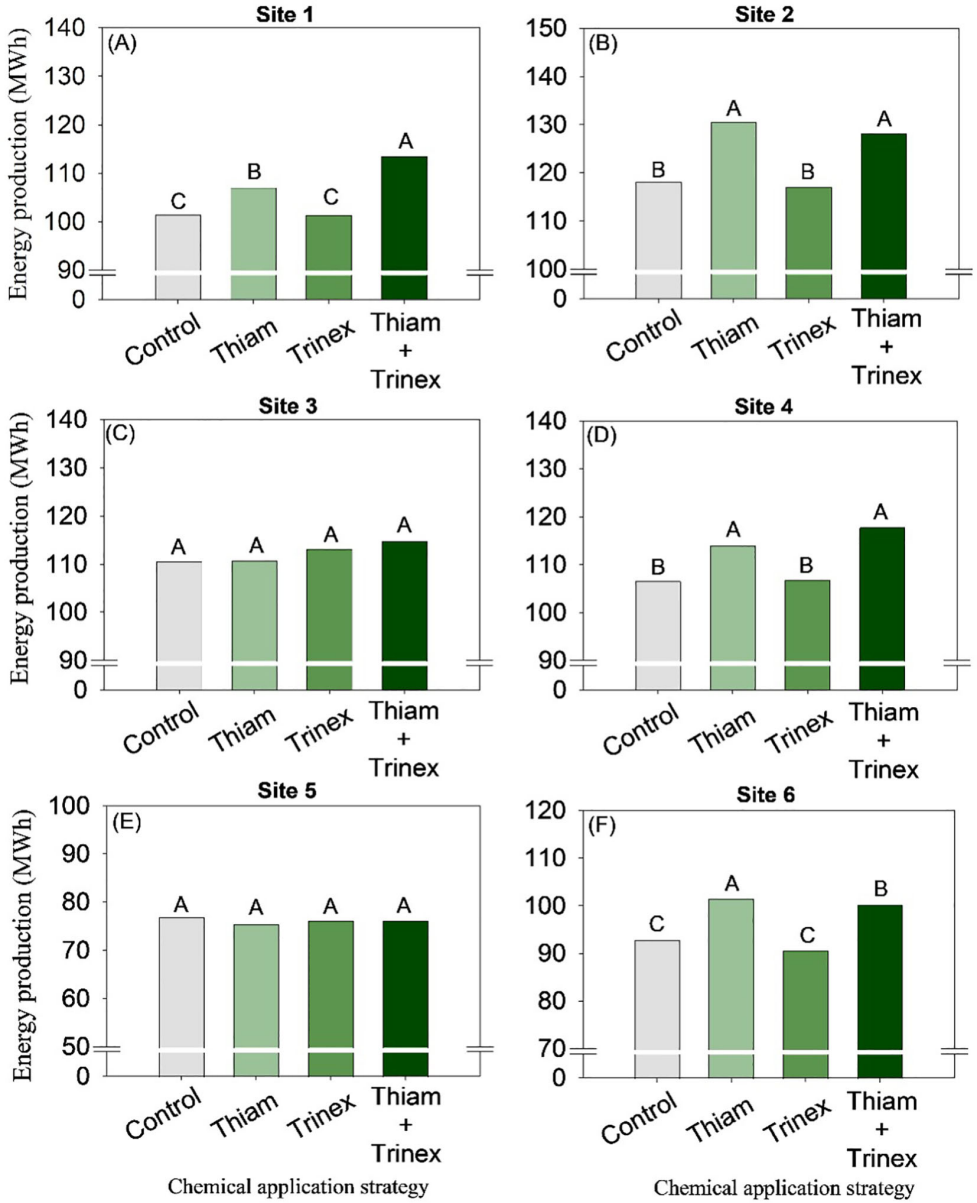
Energy production of sugarcane receiving the application of thiamethoxam as bioactivator and trinexapac-ethyl as ripener at Site 1 **(A)**, Site 2 **(B)**, Site 3 **(C)**, Site 4 **(D)**, Site 5 **(E)** and Site 6 **(F)**. Treatments of chemical application strategy mean control, no-chemical applications; Thiam, thiamethoxam applied at 60 after sugarcane regrowth stage; Trinex, trinexapac-ethyl applied at 45 days before sugarcane harvest; and Thiam+Trinex, thiamethoxam and trinexapac-ethyl applied as treatments Thiam (60 after sugarcane regrowth stage) and Trinex (45 days before sugarcane harvest). All data show the means of four replicates. Different letters indicate significant (p < 0.05) differences between treatments by LSD test.

On average, bagasse and trash yield were higher in the treatments in which thiamethoxam was applied alone or in association with trinexapac-ethyl (**Figures 8**, **9**). These increases were directly proportional to the increases in stalk yield and, to a lesser extent, fiber. The increase in bagasse production in thiamethoxam and thiamethoxam + trinexapac-ethyl reached 1.1 Mg ha^-1^ at site 6 compared with the control (11.9 Mg ha^-1^). Energy production (**Figure 10**) followed the same pattern as trash yield, as these parameters are directly proportional, the greater the amount of trash, the higher the energy production. In early harvest conditions, only site 3 showed no significant differences among treatments. The application of thiamethoxam combined with trinexapac-ethyl resulted in higher energy production, with an average increase of 13% compared to the control (108.7 MWh). In late harvest, treatment effects were observed only at site 6 (**Figure 10F**). Unlike early harvest results, the application of thiamethoxam alone led to a 16.8% increase in energy production compared to the control (92.9 MWh).

### 4.4 Pearson correlation

**Figure 11:**
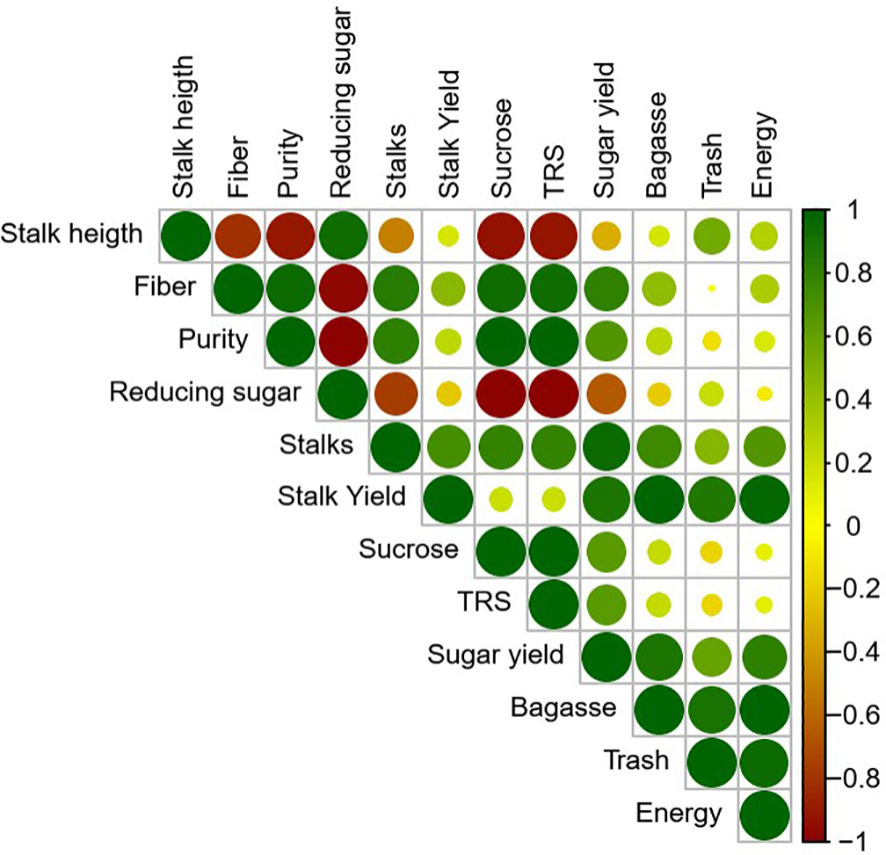
Heatmap of Pearson correlation coefficients covering all variables shown.

Significant Pearson correlations were observed among the morphophysiological, technological, and yield-related variables of sugarcane (**Figure 11**). Stalk yield showed a strong positive correlation with the number of stalks (r > 0.9), indicating that stalk density is a key determinant of biomass accumulation. This variable was also positively correlated with sugar yield, bagasse, trash, and energy production, highlighting that increases in plant productivity directly enhance industrial efficiency. Conversely, higher levels of fiber and reducing sugars were negatively correlated with technological quality parameters such as purity, sucrose concentration, and total recoverable sugar (TRS), suggesting that these components negatively affect the extraction and industrial processing of raw material. Additionally, a strong positive correlation was found among sucrose content, TRS, and sugar yield, reinforcing the interdependence of these variables in determining the technological potential of the crop.

### 4.5 Exploratory analysis: hydroclimatic influence

An exploratory analysis of the hydroclimatic conditions (**Figure 1**) and their association with sugarcane agronomic performance revealed potential patterns across sites. The highest stalk and sugar yields observed at Site 1 (early season) and Site 6 (late season) coincided with periods of elevated rainfall and air temperatures. Despite these unfavorable conditions, treatments with thiamethoxam, alone or in combination with trinexapac-ethyl, promoted significant increases in stalk yield and energy output compared to the control, suggesting a compensatory effect.

Although direct regression or climate-yield correlation analyses were not included, the consistency of thiamethoxam’s positive effect under high hydroclimatic variability supports its potential as a physiological enhancer in stress-prone environments. These findings highlight the importance of integrating climatic data into crop management studies and underscore the need for future analyses combining climatic variables with agronomic responses to better quantify these interactions.

## 5 Discussion

Sugarcane efficiently utilizes sunlight for C_4_ photosynthesis and consequently has high rates of photosynthesis and photorespiration (Sage et al., 2012; Marquardt et al., 2021). Photosynthetic efficiency and, consequently, plant growth and sugar accumulation are directly related to solar radiation. Decreases in incident light (from 15% to 20%) from solar radiation are translated by the plant into thinner stalks, narrow green-yellowish leaves, and decreased production of dry matter (Aude, 1993; Feng et al., 2019; Yang et al., 2021).

To maintain regular growth metabolism and satisfactory development, sugarcane needs high levels of sunlight. For instance, in the present study, sucrose concentrations in sugarcane might be lower than 13% at some sites due to greater rainfall in the early and late season, respectively, and higher temperatures (**Figure 1**). These conditions may be related to an increased the plants’ respiration rate and thus the demand for carbohydrates; in addition, the greater number of rainy days decreased solar incidence on the plant canopy, directly affecting photosynthesis and resulting in lower values of sucrose content.

Sucrose concentration is an important indicator of sucrose content in sugarcane when correlated with Brix (soluble solids) and RS (reducing sugars). These parameters permit an estimate of the ideal stage of ripening for sucrose accumulation in stalks and purity of the sugarcane juice (Fernandes, 2011), which facilitates planning and agro-industrial utilization of sugarcane. Purity above the minimum threshold for industrial processing of 80% (Silva et al., 2022; Jacomassi et al., 2024; Mehdi et al., 2024) increases industrial yield.

Upon absorption by leaves, ripener is translocated to meristematic regions of rapid growth. Trinexapac-ethyl acts directly on the synthesis of the plant hormone gibberellic acid (GA) by temporarily inhibiting the conversion of the precursor of this hormone (GA20). By preventing 3-β-hydroxylation, trinexapac-ethyl prevents the synthesis of GAs with high biological activity after formation of GA12. Reducing the levels of GA1, which is one of the most biologically active GAs and efficiently enables cell elongation, decreases the demand from sinks for metabolic energy for growth (Resende et al., 2000; Rodrigues et al., 2018). In addition, inhibiting GA synthesis induces sucrose accumulation in the stalks and consequently elevates sucrose content. Similarly positive effects of trinexapac-ethyl application on these qualitative parameters of sugarcane were reported previously by others (Leite and Crusciol, 2008; Leite et al., 2010, 2015a).

The positive effects of ripeners are greatest when applied under environmental conditions favorable to vegetative development of the crop, such as those generally observed in the early and late harvest seasons. In the absence of ripener application, sugarcane quality was reduced under unfavorable conditions of low water availability and/or low temperature.

At sites 4 and 5, sucrose concentration levels in the control treatment were below the minimum sucrose content (13%) recommended for sugarcane industrialization (Castro et al., 2008) due to unfavorable climatic conditions for natural ripening. These findings highlight the necessity of agronomic management practices that mitigate negative effects of climate on sugarcane maturation. Ripener use also impacted sucrose concentration by decreasing the proportion of reducing sugars (glucose and fructose). This shift in sugar composition reflects the suppression of apical elongation and the formation of shorter internodes. Although these changes could potentially reduce the plant weight of the analyzed samples, stalk yield and sugar production were not affected (Viana et al., 2007, 2008, 2017a; Leite et al., 2009a).

In addition, no response of fiber to ripener application was observed in the present study, corroborating the results of several previous studies (Caputo et al., 2008; Leite et al., 2009a, 2009b, 2010). However (Guimarães et al., 2005), observed negative effects of trinexapac-ethyl on fiber content throughout the sampled seasons, and other ripeners with different mechanisms of action have also been reported to reduce fiber content (Dalley and Richard, 2010; Alvarez et al., 2016; Karmollachaab et al., 2016; Viana et al., 2017b, 2017a).

The mode of action of both trinexapac-ethyl and thiamethoxam is likely related to the lack of significant effects on fiber content. Trinexapac-ethyl, by inhibiting gibberellin synthesis, reduces internode elongation in sugarcane and promotes sucrose accumulation in the stalks (Caputo et al., 2008; Leite et al., 2009b; Dalley and Richard, 2010). However, this mechanism does not directly affect the structural composition of the cell wall, such as the deposition of lignin and cellulose, which are the primary components of plant fiber.

Thiamethoxam, on the other hand, acts as a bioactivator, enhancing physiological responses such as those related to tolerance to biotic and abiotic stresses, thereby increasing biomass production and crop productivity. Nevertheless, these effects are associated with general plant metabolism and growth dynamics, rather than modifications in cell wall composition (Vieira et al., 2014; Blois Villela et al., 2015; House et al., 2021). As a result, fiber content remains unaffected, as it is largely governed by genetic factors that are not altered by the application of either the ripener or the insecticide.

Indeed, the improvements in sugarcane development and yields may be due to the potential phytotonic effects of trinexapac-ethyl and thiamethoxam. Sugarcane producers frequently report that after the application of thiamethoxam, plants exhibit increased vigor and development. In Brazil, this insecticide is widely used to control *Mahanarva fimbriolata* (root froghopper) and *Heterotermes tenus* (termites). Although its primary function is as an insecticidal agent, previous studies, including the present investigation, indicate that thiamethoxam also has bioactivating effects. These effects may be related, among other factors, to the indirect induction of endogenous hormone synthesis, raising interest in its agricultural use to enhance agronomic parameters (Castro and Pereira, 2008; Pereira et al., 2010; Martins et al., 2012).

Several studies suggest that neonicotinoid insecticides, including thiamethoxam, may influence gene expression in plants, potentially eliciting biostimulant effects that enhance tolerance to both biotic and abiotic stresses. In agricultural crops, thiamethoxam has been associated with pathways involved in the biosynthesis of key phytohormones such as abscisic acid (ABA), auxin (IAA), and gibberellins (GA) (Ford et al., 2010; Almeida et al., 2012; Stamm et al., 2014; Afifi et al., 2015; House et al., 2021). It possible may include those regulated by genes like NCED, YUCCA, and GA20ox (Cao et al., 2019; Gavassi et al., 2021; Yue et al., 2023). However, the precise molecular mechanisms remain to be fully elucidated, these associations point to a possible role of thiamethoxam in modulating hormone-related signaling networks.

In addition to these hormones, the use of neonicotinoids has also been correlated with increased concentrations of salicylic acid (SA), jasmonic acid (JA), and cytokinins (CK). These phytohormones play a central role in regulating key physiological processes such as drought tolerance, stomatal aperture control, and the activation of systemic acquired resistance (SAR) (Ford et al., 2010; Szczepaniec et al., 2013; Taiz et al., 2017).

Interestingly, many of these hormonal pathways are intimately connected to amino acid metabolism. For instance, the biosynthesis of indole-3-acetic acid (IAA) via the YUCCA pathway is derived from tryptophan, a key aromatic amino acid. Similarly, JA and SA signaling can be influenced by the availability of amino acids such as methionine and phenylalanine, which serve as precursors or modulators of these defense-related hormones (Almeida et al., 2012; Macedo et al., 2013; Li et al., 2024). Thus, the action of thiamethoxam in promoting hormone biosynthesis may also reflect broader metabolic shifts in amino acid pathways, suggesting a coordinated regulatory network where gene expression, hormonal balance, and nitrogen metabolism converge to optimize plant growth and stress resilience. Thus, the sum of these responses may result in agronomic benefits, such as enhanced crop productivity.

These physiological changes triggered by thiamethoxam highlight its function as a plant bioactivator. Such effects include, among others, improved root development, optimization of stomatal conductance, and activation of hormone-mediated defense mechanisms. As demonstrated in our study (**Figures 4** and **7**), these processes are directly associated with increased stalk and sugar yield per hectare.

As mentioned, the insecticide enhances the efficiency of roots in their specific functions, i.e., the fixation, absorption and transport of water and mineral nutrients, resulting in greater tiller survival and stalk numbers at harvest. On the other hand, studies have shown that applying trinexapac-ethyl as a ripener improves sugarcane ratoon regrowth compared with control treatment and, consequently, the number of stalks at harvest of the subsequent crop (Leite et al., 2011; Martins et al., 2012).

It probably explains, in general, why the metabolic effects of thiamethoxam had different impact on plant height and number of stalks, both related to biometric measurements. The joint effect of these two chemicals is more related to boost the quality and longevity of the ratoon sprot rather than plant’s height itself.

Also, because when ripener is applied under conditions favorable to growth and unfavorable for natural ripening, plant height is decreased because of trinexapac-ethyl on endogenous levels of active forms of GA, which leads to reduced stalk elongation due to the direct links of this hormone with growth and cell division (Tymowska-Lalanne and Kreis, 1998; Resende et al., 2003; Taiz et al., 2017).

In addition, thiamethoxam may increase stalk yields by promoting the activation or repression of the transcription and/or expression of certain plant genes, thereby promoting the action of metabolic enzymes and membrane proteins that favor the uptake of water and nutrients (Castro et al., 2008; Macedo and Castro, 2011). Thiamethoxam bolsters plant stress defense mechanisms, increasing the plant’s ability to face adverse conditions and inducing morphophysiological changes that may result in better plant development (Eibner, 1986; Castro et al., 2008; Li et al., 2022; Zhao et al., 2023).

Additionally, we have observed in our study that the application of trinexapac-ethyl alone did not influence stalk number or yield and enhanced only qualitative parameters. However, the increase in sugar yield in the treatments with trinexapac-ethyl is directly related to the product of sucrose content and stalk yield (Leite et al., 2009a, 2009b). A slight decrease in stalk yield can result in considerable increases in the quality of the raw material, i.e., increases in tons of sugar per hectare, as the ripener molecule primarily suppresses vegetative growth when its application is appropriately managed (van Heerden, 2014b; van Heerden et al., 2015).

All these benefits were also reflected in the energy cogeneration capacity of sugarcane mills, since the production of bagasse and trash is not determined solely by the genetic characteristics of different varieties but is also strongly influenced by crop productivity and stalk production — parameters that were significantly enhanced using thiamethoxam. Fiber and stalk yield can be used to calculate bagasse at 50% moisture, and trash yield is calculated considering 140 kg of trash per Mg of stalk (Hassuani, 2005). In other words, the fact that the increase in energy productivity is directly linked to the gains in biomass accumulation in the field, summarize that our raising energy results (**Figure 10**) are directly related to the thiamethoxam applied on sugarcane, even in association with ripener use.

In addition to our results, it is relevant to discuss some other specific points related to the thiamethoxam. First, to specifically isolate and evaluate the bioactivating effect of thiamethoxam, the experiment was conducted in areas with low pest incidence. This approach aimed to prevent potential interference from biotic damage caused by insects, which could compromise the analysis of agronomic parameters.

It is essential to consider that plants exposed to biotic stress factors, such as herbivorous insect attacks, undergo significant biochemical changes. Among these changes, the increased production of reactive oxygen species (ROS) stands out, potentially leading to oxidative stress (Nascimento and Barrigossi, 2014; Du et al., 2024). Additionally, morphological modifications and symptoms such as chlorosis, necrosis, poor tiller formation, and premature senescence are observed, among other responses associated with biotic stress conditions (Goggin, 2007; Gimenez et al., 2018; González Guzmán et al., 2022; Du et al., 2024). Thus, such physiological and morphological alterations could impact the results of the present study, reinforcing the need to evaluate the effects of thiamethoxam under controlled conditions, free from direct pest influence.

The data obtained in our study confirm that even in low insect incidence conditions in the field, thiamethoxam exerts a positive phytotonic effect, promoting greater plant vigor, development, and productivity. However, considering that this molecule was originally developed as an insecticide, there is concern that its bioactivating action may be influenced by the phytosanitary context of the crop. Therefore, generalizing the observed positive effects requires caution, particularly in scenarios where the crop is under low pest pressure.

In addition to the phytosanitary context, environmental conditions, particularly the hydroclimatic balance, may also modulate the effectiveness of thiamethoxam as a biostimulant. Hydroclimatic conditions, especially the interaction between rainfall and temperature, play a critical role in sugarcane development. Meteorological data (**Figure 1**) revealed that early and late harvest periods were characterized by higher rainfall and temperatures, which can impair sucrose accumulation due to increased respiration and reduced photosynthesis. Interestingly, under these suboptimal ripening conditions, thiamethoxam consistently improved stalk yield and energy production (**Figures 4**, **10**), suggesting a mitigating effect against climatic stress. These findings reinforce the hypothesis that thiamethoxam’s bioactivating effect may be enhanced under hydroclimatic imbalance, likely due to its role in hormone signaling and stress tolerance pathways (Ford et al., 2010; House et al., 2021). Although our correlation analysis focused on agronomic traits (**Figure 11**), future regression-based approaches could further clarify the interaction between thiamethoxam and climatic variables, enabling more precise management recommendations.

To contextualize these physiological and agronomic effects, it is important to also consider the chemical nature, systemic behavior, and regulatory implications associated with thiamethoxam use in sugarcane cultivation. Given this, future studies should further explore the impacts of thiamethoxam under different levels of pest infestation. Investigations correlating the metabolic increase promoted by this compound with the plant’s response to biotic stress could contribute to a more comprehensive understanding of its bioactivating potential. Furthermore, such studies will allow for an assessment of the effects of this mechanism on productivity parameters and raw material quality in sugarcane cultivation.

Another relevant topic to discuss is the fact that thiamethoxam is a systemic insecticide belonging to the neonicotinoid class, specifically within the nitroguanidine subgroup. Its mode of action involves interaction with nicotinic acetylcholine receptors on insect neural membranes, disrupting nerve impulse transmission and leading to the organism’s death.

Despite its effectiveness in pest control, the use of neonicotinoids has been widely debated due to their negative impact on non-target organisms, particularly pollinators. In Brazil, the Brazilian Institute of Environment and Renewable Natural Resources (IBAMA) imposes restrictions on the application of thiamethoxam to mitigate environmental risks associated with its use.

In sugarcane cultivation, its application is permitted under strict regulations, aiming to balance agronomic benefits with the reduction of ecological impacts. In this context, integrated pest management practices, such as pest population monitoring and the use of biological control agents, are essential strategies to minimize dependence on insecticides and mitigate potential environmental damage.

All regulatory guidelines and policies regarding the use of thiamethoxam in sugarcane cultivation, including aspects related to application timing restrictions, dosage and frequency limitations, mandatory mitigation measures, agronomic justifications, and phased risk assessments, are detailed in the Final Technical Report – SEI IBAMA No. 17732614, which consolidates the environmental analysis conducted by the regulatory agency (Instituto Brasileiro do Meio Ambiente e dos Recursos Naturais Renováveis (IBAMA, 2024).

Also, over the past decades, the intensive use of neonicotinoids has been associated with the emergence of resistance mechanisms in several insect pest species of agricultural importance. This phenomenon has been extensively documented through studies aiming to elucidate the location and function of metabolic targets involved in resistance to this class of insecticides. Notable examples include *Drosophila melanogaster*, *Nilaparvata lugens*, *Myzus persicae*, and *Aphis gossypii* (Gorman et al., 2008; Bass et al., 2011; Hirata et al., 2017; Homem et al., 2020; Matsuda et al., 2020).

Regionally, in Brazil, *Bemisia tabaci* (whitefly) has been identified as an important insect species exhibiting resistance to thiamethoxam (Silva et al., 2009; Esashika et al., 2016). However, to date, there are no documented cases of resistance in *Mahanarva fimbriolata*, the primary target of this neonicotinoid in sugarcane cultivation.

In general, resistance mechanisms are linked to enhanced metabolic detoxification processes in insects, primarily mediated by cytochrome P450 monooxygenases (CYPs). Additionally, modifications in the nicotinic acetylcholine receptor (nAChR) have been observed, reducing the insecticide’s binding affinity and thereby compromising its effectiveness (Scott, 1999; Liu et al., 2005; Nauen et al., 2022; Xu et al., 2022).

Although the present study does not directly assess insect resistance parameters, it is important to consider that prolonged use of neonicotinoid compounds may exert selective pressure on pest populations, potentially leading to resistance over time. Therefore, the adoption of integrated pest management (IPM) practices is essential. These include the rotation of insecticides with different modes of action, the use of biological control methods, and regular pest monitoring.

Furthermore, guidelines established by the Brazilian Institute of Environment and Renewable Natural Resources (IBAMA) aim to systematically implement practices that help mitigate the long-term impacts of pest resistance, promoting the sustainable use of insecticides in agricultural systems.

Therefore, the use of thiamethoxan and trinexapac-ethyl in association promotes a desirable balance between the industrial quality of sugarcane juice and stalk yield per hectare, resulting in a greater volume of biomass at harvest time. This combination increases the quality of the raw material and is an alternative crop management strategy that may increase economic gains. Despite the evident bioactivating effect of thiamethoxan, it is important to highlight that its use should be restricted to pest control, in the present’s regulatory conditions, since it is an insecticide, but thiamethoxan could be used for pest control in areas where the ripener trinexapac-ethyl is applied.

## 6 Conclusion

The commonly applied insecticide thiamethoxam has phytotonic effects on sugarcane, as evidenced by the enhanced biometric parameters of early and late harvest sugarcane in this study. Due to its bioactivating effects, thiamethoxam benefited sugarcane development, with increases in number of stalks and yield as well as energy, trash, and bagasse production. The isolated and/or combined application of thiamethoxam with trinexapac-ethyl as a ripener increased sugar yield at sites 1, 2, 4, and 6. In contrast, the application of trinexapac-ethyl alone or in combination enhanced sugar yield across all evaluated sites. Importantly, the biometric gains due to the combination of thiamethoxam and trinexapac-ethyl did not reduce industrial quality (technological parameters).

Furthermore, there is a sustainability-driven perspective in which the integration of thiamethoxam into crop management practices may promote more efficient land use. However, as it is a compound classified as a neonicotinoid, further research is required to ensure safety standards regarding its prolonged use in sugarcane production systems. Such studies are essential to establish a solid technical foundation that supports sustainable agricultural practices, balancing high productivity with environmental responsibility.

## Data Availability

The original contributions presented in the study are included in the article/supplementary material. Further inquiries can be directed to the corresponding author.
