# Supplementary material for: Effects of thiamethoxam insecticide on sugarcane plant growth under chemical ripening at early and late harvest
Source: Front Plant Sci. 2025 Jun 17;16:1558071. doi: 10.3389/fpls.2025.1558071 (PMC12210020; doi:10.3389/fpls.2025.1558071)
Supplement: Supplementary file 1 [file Table1.docx]

Supplementary Table. P values indicate the effect of sugarcane receiving the application of thiamethoxam as bioactivator and trinexapac-ethyl as ripener at ratoon regrowth in all parameters analysis.

| Treatments | Early Harvest Season | | | |  | Late Harvest Season | |
| --- | --- | --- | --- | --- | --- | --- | --- |
|  |  |  |  |  |  |  |  |
|  | Site 1 | Site 2 | Site 3 | Site 4 |  | Site 5 | Site 6 |
|  | Sucrose Concentration (%) | | | | | | |
|  |  |  |  |  |  |  |  |
| P value | *0,015* | *0,011* | *0,023* | *<0,001* |  | *0,049* | *0,037* |
|  |  |  |  |  |  |  |  |
|  | Purity (%) | | | | | | |
|  |  |  |  |  |  |  |  |
| P value | *0,037* | *0,036* | *0,044* | *0,025* |  | *0,021* | *0,039* |
|  | Fiber (%) | | | | | | |
|  |  |  |  |  |  |  |  |
| P value | *0,158* | *0,203* | *0,338* | *0,174* |  | *0,150* | *0,189* |
|  | Reducing Sugars (%) | | | | | | |
|  |  |  |  |  |  |  |  |
| P value | *0,012* | *0,023* | *0,014* | *0,037* |  | *0,049* | *0,042* |
|  | Total Reducing Sugars Kg Mg^-1^ | | | | | | |
|  |  |  |  |  |  |  |  |
| P value | 0,010 | 0,015 | 0,019 | <0,001 |  | 0,037 | 0,022 |
|  | Stalks m^-1^ | | | | | | |
|  |  |  |  |  |  |  |  |
| P value | *0,009* | *0,015* | *0,227* | *0,029* |  | *0,163* | *0,024* |
|  | Stalks Height m | | | | | | |
|  |  |  |  |  |  |  |  |
| P value | *0,049* | *0,042* | *0,188* | *0,202* |  | *0,156* | *0,033* |

|  | Stalk Yield Mg ha^-1^ | | | | | | |
| --- | --- | --- | --- | --- | --- | --- | --- |
|  |  |  |  |  |  |  |  |
| P value | *0,003* | *0,026* | *0,515* | *0,020* |  | *0,778* | *0,001* |
|  | Bagasse | | | | | | |
|  |  |  |  |  |  |  |  |
| P value | 0,002 | 0,021 | 0,536 | 0,026 |  | 0,635 | <0.001 |
|  | Trash | | | | | | |
|  |  |  |  |  |  |  |  |
| P value | 0,022 | 0,027 | 0,651 | 0,028 |  | 0,697 | <0.001 |
|  | Energy | | | | | | |
|  |  |  |  |  |  |  |  |
| P value | 0.032 | 0.002 | 0,596 | 0.021 |  | 0.764 | 0.015 |

^*^Significant *p≤*0.05.
